# Binding Behavior of Human Hepatoma-Derived Growth Factor on *SMYD1*

**DOI:** 10.1021/acs.jpcb.4c01854

**Published:** 2024-08-02

**Authors:** Jan-Kai Wu, Ying-ying Lee, Hsin Hung, Yuan-Ping Chang, Ming-Hong Tai, Hsiu-Fang Fan

**Affiliations:** † Institute of Medical Science and Technology, National Sun Yat-sen University, Kaohsiung 80424, Taiwan; ‡ Department of Chemistry, National Sun Yat-sen University, Kaohsiung 80424, Taiwan; § Aerosol Science Research Center, National Sun Yat-sen University, Kaohsiung 80424, Taiwan; ∥ Institute of Biomedical Science, National Sun Yat-sen University, Kaohsiung 80424, Taiwan

## Abstract

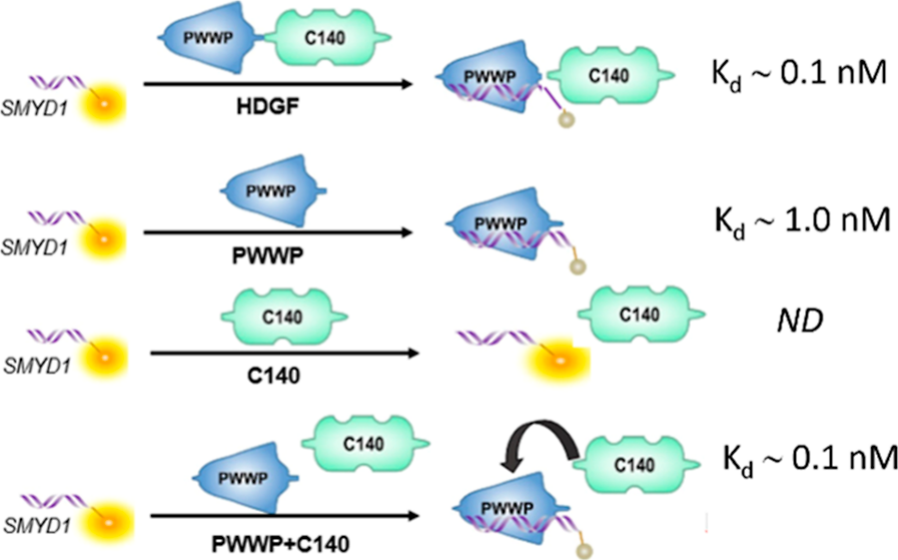

The protein-induced fluorescence change technique was employed to investigate the interactions between proteins and their DNA substrates modified with the Cy3 fluorophore. It has been reported that the human hepatoma-derived growth factor (HDGF), containing the chromatin-associated N-terminal proline–tryptophan–tryptophan–proline (PWWP) domain (the N-terminal 100 amino acids of HDGF) capable of binding the *SMYD1* promoter, participates in various cellular processes and is involved in human cancer. This project investigated the specific binding behavior of HDGF, the PWWP domain, and the C140 domain (the C-terminal 140 amino acids of HDGF) sequentially using protein-induced fluorescence change. We found that the binding of HDGF and its related proteins on Cy3-labeled 15 bp *SMYD1* dsDNA will cause a significant decrease in the recorded Cy3 fluorophore intensity, indicating the occurrence of protein-induced fluorescence quenching. The dissociation equilibrium constant was determined by fitting the bound fraction curve to a binding model. An approximate 10-time weaker *SMYD1* binding affinity of the PWWP domain was found in comparison to HDGF. Moreover, the PWWP domain is required for DNA binding, and the C140 domain can enhance the DNA binding affinity. Furthermore, we found that the C140 domain can regulate the sequence-specific binding capability of HDGF on *SMYD1*.

## Introduction

The hepatoma-derived growth factor (HDGF, uniport ID: P51858) is a novel growth factor initially identified in the culture medium of human hepatoma-derived HuH-7 cells.^1^ It has been reported to stimulate the proliferation of liver cancer cells and is highly expressed in various human cancer tissues.^2^ HDGF is linked to several cancer characteristics, including rapid growth, invasion, and metastasis.^3^ It promotes cell proliferation through multiple signaling pathways, such as activating mitogen-activated protein kinase and phosphatidylinositol 3-kinase, which leads to increased production of growth factors and regulation of nuclear protein target gene expression.^4^

Structurally, HDGF comprises two domains: an N-terminal domain featuring a proline–tryptophan–tryptophan–proline (PWWP) motif (residues 1–100 in human HDGF) and a variable C-terminal domain (residues 101–240, C140).^5^ The PWWP domain, located at the N terminus of HDGF,^1^ consists of a 90-amino acid sequence characterized by a conserved PWWP core. This motif is found in over 60 eukaryotic proteins.^6−8^ HDGF’s interaction with nucleolin (NCL) facilitates its nuclear translocation.^9^ This protein has been shown to specifically bind to the *SMYD1* promoter, isolated through the chromatin immunoprecipitation (ChIP) method.^10^ Moreover, the binding of HDGF to DNA requires a conserved amino acid sequence known as the PWWP motif.^6,10^

Moreover, HDGF serves as a mitogen across various cell types, with its nuclear localization being pivotal for promoting cell division.^11^ This role is modulated by post-translational modifications, particularly phosphorylation at residue 103, which significantly influences its mitogenic activity. Phosphorylation at S103 is essential for regulating HDGF’s function: the S103A mutation results in the loss of mitogenic activity, whereas the S103D phospho-mimic mutation increases this activity compared to the wild-type HDGF.^12^ However, the specific function of phospho-S103-HDGF during mitosis is not yet fully understood. Despite the unclear mechanism behind HDGF’s stimulation of cell proliferation following nuclear translocation, there is evidence suggesting that HDGF binds to target gene promoters, thereby affecting DNA transcription.^12^ This raises an important question: could phosphorylation within HDGF regulate its DNA-binding process? Could such regulation involve inducing conformational changes in the protein or enhancing its interactions with chromatin-binding proteins?

Using NMR titration, the PWWP domain of HDGF has been determined to exhibit nonspecific DNA-binding behaviors.^13^ Moreover, the PWWP domain of human mismatch repair (MMR) protein MSH6 has been reported to have a stronger binding affinity for double-stranded DNA (dsDNA) over single-stranded DNA (ssDNA) with a dissociation equilibrium constant, *K*_D_, of approximately nM.^14^ Dissociation equilibrium constants, *K*_D_, of approximately 8 and 230 nM have also been reported for Δ218 and the PWWP domain of mammalian DNA methyltransferase Dnmt3b, respectively.^7^ Distinct DNA-binding behaviors have been observed among PWWP domains in various proteins. For instance, the PWWP domains of DNMTB and HDGF show nonspecific DNA interactions, whereas those of LEDGF and HRP3 display specific sequence preferences.^7,13−16^ Most of these dissociation equilibrium constants, *K*_D_, were determined from the electrophoresis mobility shift assay (EMSA),^14,15^ nitrocellulose filter-binding assay,^7^ and surface plasmon resonance.^17^ Protein-induced fluorescence enhancement (PIFE) is a photophysical phenomenon typically observed in fluorescent dyes belonging to the cyanine family, such as the Cy3 fluorophore. Studies suggest that the presence of proteins reduces the rate of cis–trans photoisomerization. This is likely due to the proteins affecting the rotational freedom of the fluorophore, thereby influencing its fluorescence intensity.^18^ Therefore, PIFE can be employed to study protein–DNA interactions by measuring fluorescence intensity to determine protein binding constants, substrate specificity, and kinetics.^19^ Recently, it has been noted that protein binding can result in not only fluorescence enhancement (PIFE) but also fluorescence quenching (PIFQ).^20^ In this study, we investigated the specific binding behavior of HDGF, the PWWP domain, the C140 domain, and S103A using a protein-induced fluorescence change technique to determine the dissociation equilibrium constant. By employing the aforementioned assay, we were able to elucidate the binding behaviors, substrate preferences, and regulation mechanisms of HDGF in DNA binding.

## Materials and Methods

### Proteins and DNA Substrates

**Figure 1:**
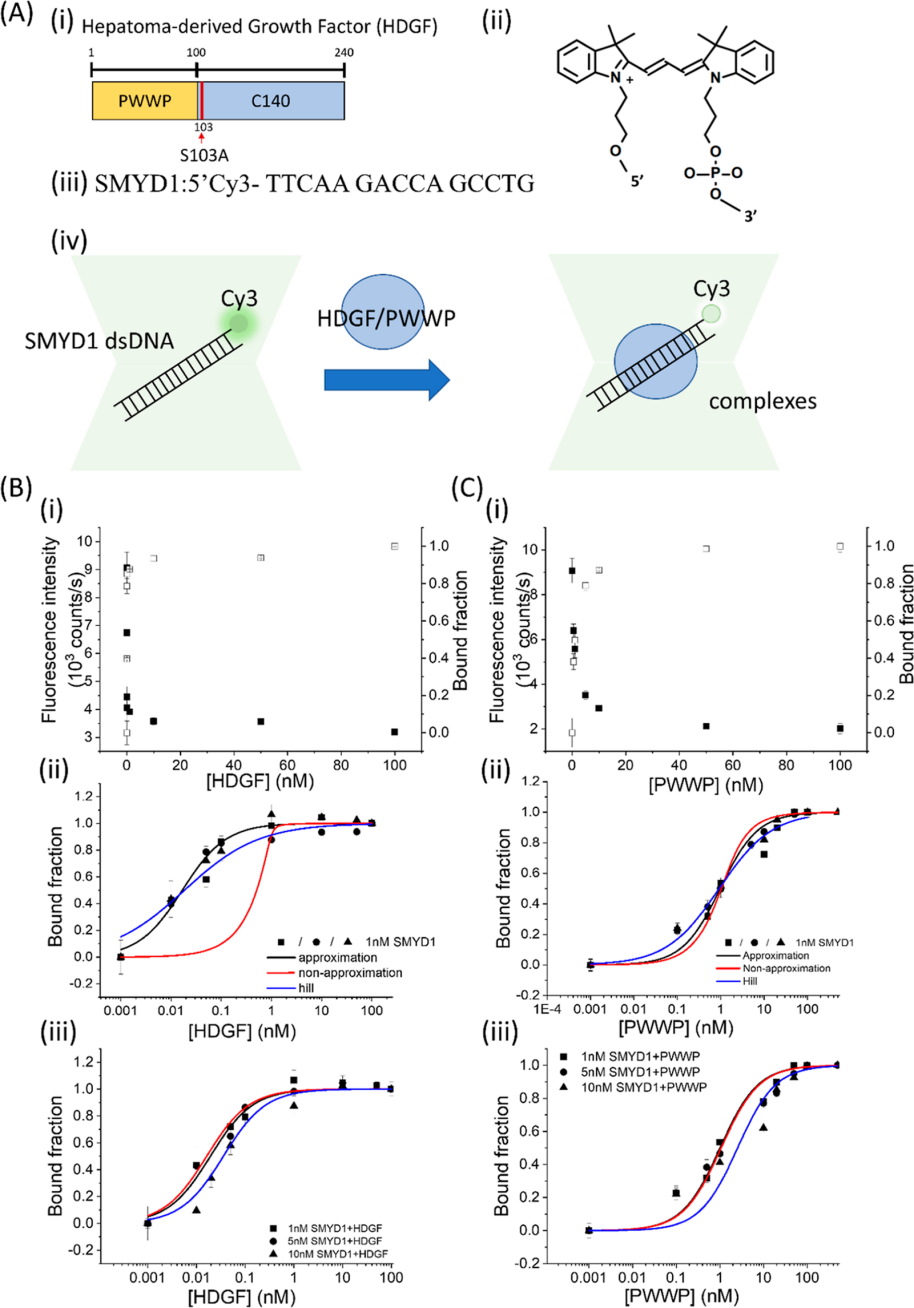
Protein-induced fluorescence changes to investigate SMYD1 dsDNA binding affinity. (A) (i) Domain architecture of HDGF and the mutants used in this experiment. (ii) Structure of the Cy3 fluorophore. (iii) Sequence of *SMYD1*. (iv) Schematic of HDGF binding to a Cy3-labeled duplex DNA signaled by significant fluorescence intensity quenching (PIFQ). (B) Protein-induced fluorescence quenching to investigate binding affinity of HDGF to *SMYD1* fitted with different models. (i) Fluorescence changes of 1 nM Cy3-labeled *SMYD1* in response to various concentrations of HDGF and the corresponding bound fraction. The solid circles indicate the fluorescence intensity. The open squares indicate the normalized bound fraction. (ii) Three repeated experiments to investigate the interaction between HDGF and 1 nM *SMYD1* fitted with three binding models. (iii)Three representative experiments to investigate the interaction between HDGF and various concentrations of *SMYD1* (1, 5, and 10 nM) fitted with three binding models. (C) Protein-induced fluorescence change to investigate binding affinity of the PWWP domain to various concentrations of SMYD1 fitted with different models. (i) Fluorescence changes of 1 nM Cy3-labeled SMYD1 in response to various concentrations of the PWWP domain and the corresponding bound fraction. The solid circles indicate the fluorescence intensity. The open squares indicate the normalized bound fraction. (ii) Three repeated experiments to investigate the interaction between the PWWP domain and 1 nM *SMYD1* fitted with three binding models. (iii) Three representative experiments to investigate the interaction between PWWP and various concentrations of *SMYD1* (1, 5, and 10 nM) fitted with three binding models. The lines represent the fitting curves with the Hill equation fitting model (blue: —), approximation fitting model (black: —), and nonapproximation model (red: —) to obtain corresponding dissociation equilibrium constants, *K*_D_, listed in Table 1.

The HDGF and its related mutants, PWWP, C140 domain, and S103A, were expressed in *Escherichia coli* and purified according to previously published procedures.^4^ The human HDGF gene was replicated from a human fetal brain cDNA library using PCR, as previously described.^21^ The PCR-amplified HDGF, PWWP domain, C140 domain, and S103A were then separately inserted into the pET28a vector and introduced into *E. coli* BL21-Codon Plus-RIL for the production and purification of recombinant HDGF and its mutants. All constructed plasmids were verified by DNA sequencing. DNA oligonucleotides were custom-designed and purchased from a local supplier (MDBio, Inc. Taiwan). DNA sequences are listed in Table 1. A previous study reported that the PWWP domain can form complexes with various lengths of *SMYD1*, ranging from 5 to 15 bp.^4^ In this study, the 15 bp *SMYD1* sequence, 5′-TTCAAGACCA GCCTG, was selected to investigate the binding behaviors of HDGF and its mutants. The structures of the Cy3 fluorophores are shown in Figure 1A.

**Table 1:**
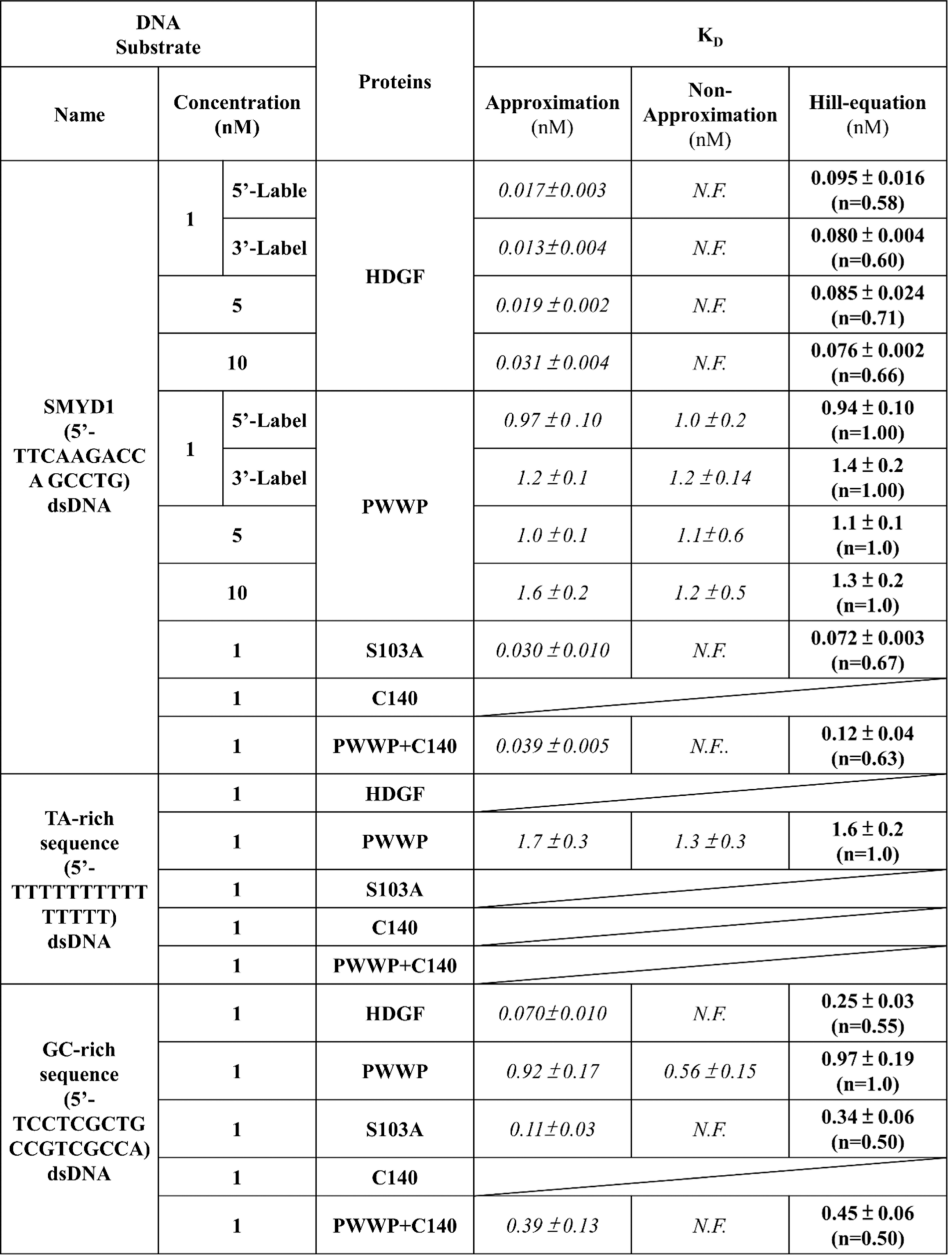
Dissociation Equilibrium Constants *K*_D_ of HDGF and Relative Proteins to dsDNA Molecules^a^

a The constants were determined by fitting with the Hill equation fitting model, approximation fitting model, and nonapproximation model. The *K*_D_ values presented in Table 1 were derived from these three models, all with adjusted *R*-square values >0.8. The fitting results with adjusted *R*-square values <0 were indicated with N.F., meaning cannot be fitted. The cell contains a diagonal line indicating no detectable signal, meaning N.D., not detectable.

### Protein Purification

The expression and purification of full-length HDGF, its mutants, and truncated proteins were carried out following previously described procedures.^4^ The purification process for HDGF and related proteins involved using a Ni^2+^-NTA agarose column pre-equilibrated with binding buffer (20 mM Tris-HCl, 150 mM NaCl, pH 7.5). Unwanted proteins were removed using the binding buffer supplemented with imidazole (20–50 mM), while the target protein was eluted with the binding buffer containing a higher concentration of imidazole (150 mM). The protein was then concentrated using a Centricon filter (MWCO 10,000; Sartorius Vivaspin 20) and its purity was verified by SDS-PAGE (12%) followed by Coomassie Brilliant blue R-250 staining (Figure S1). The protein concentration was determined using a Bradford assay (Scientific Biotech Corp, BR01-500).

### Protein Binding-Induced Fluorescence Quenching

PIFQ was carried out on a homemade confocal system based on a Nikon Ti eclipse. A 532 nm laser was directed via a 405/488/532/635 nm dichroic mirror (Semrock, Di01-R405/488/532/635) and focused using a Nikon Apochromat 100 × NA 1.4 oil immersion objective to excite Cy3 fluorophores. Fluorescence emission was collected through a 405/488/532/635 nm notch filter (Semrock, NF03-405/488/532/635E-25) and detected by avalanche photodiodes (PicoQuant, MPD-5C5T). The DNA oligomers were labeled with Cy3 at either the 5′ end or 3′ end (Figures 1A and 3A). In previous studies, a 30 min incubation of HDGF proteins or their mutants with a DNA substrate at cold temperature was found to be sufficient for EMSA assays to determine binding behaviors,^10^ which led to the decision to use a 1 h incubation period to ensure equilibrium for PIFQ signal measurement. For PIFQ-based DNA-binding experiments, a mixture of Cy3-labeled dsDNA at concentrations of 1, 5, or 10 nM was preincubated with varying amounts of HDGF or its mutants (ranging from 10 pM to 100 nM) in complete HDGF buffer (46 mM NaCl, 0.9 mM KCl, 3.3 mM Na_2_HPO_4_, and 0.66 mM KH_2_PO_4_ from Bioshop, pH 7.4) on ice for at least 1 h before data acquisition. In our preliminary tests, we utilized a surface-bound DNA system coupled with a TIFR imaging platform to study binding behaviors. However, the observed changes in fluorescence intensity following protein addition were not significant (data not shown). This could be attributed to the hindering effect of an immobile surface. To mitigate the influence of immobile surfaces, we have adopted a confocal imaging system that requires smaller sample volumes, offers high temporal resolution (∼second), and provides improved signal sensitivity. A 100 μL sample solution was placed on a cover glass (MARIENFELD), excited with a 532 nm laser (Photop LDC, ∼60 μW), and the fluorescence signals were recorded using an avalanche photodiode (PicoQuant, MPD-5C5T) via a TCSPC system (PicoQuant, PicoHarp 300). To obtain average values, the fluorescence signals were recorded for 60 s, and each experimental condition was repeated five times. The fraction of protein-bound Cy3-labeled DNA molecules was determined by the change in fluorescence intensity following protein binding. This fraction was normalized to the maximum change in Cy3 fluorescence intensity observed at the highest protein concentration. Binding curves were generated by titrating Cy3-labeled DNA molecules with increasing concentrations of HDGF or its mutants. For determination of the dissociation equilibrium constant (*K*_D_), the fraction of bound DNA molecules (Y) was measured at a constant Cy3-labeled DNA concentration, varying the concentration of HDGF or the mutants. The fraction of protein-bound Cy3-labeled DNA molecules was then plotted against the protein concentration, and the data were analyzed using three different models (described in the following section) to calculate the *K*_D_.

### Algorithm for Fitting Models to Determine Dissociation Equilibrium Constant, *K*_D_

Three distinct models are utilized to describe the protein–DNA-binding behaviors. The first is an approximation of a single-site binding model, represented as
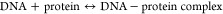
In this model, the initial DNA concentration is denoted as [D], and the protein concentration is denoted as [P]. The concentration of DNA–protein complexes formed under each condition is represented as *x*. Consequently, the dissociation equilibrium constant (*K*_D_) is expressed by the equation
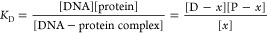


Under the pseudo-first-order approximation that the protein concentration is higher than the DNA concentration, this allows for the simplification of the dissociation equilibrium constant to
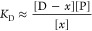


Subsequently, the relationship between the bound fraction and the dissociation equilibrium constant (*K*_D_) is illustrated by the following equations
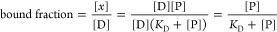
1

The second model is the nonapproximation single-site binding model. In cases where the concentration of protein is not significantly greater than the concentration of DNA, the contribution of the bound fraction, *x*, cannot be neglected. The derivation for this model is as follows
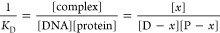


After rearrangement, this can be represented by the equation
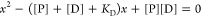


Here, *x* is the concentration of DNA–protein complexes, as described previously. This quadratic equation can be solved, leading to the quadratic formula
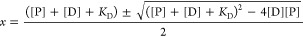


When considering the realistic, non-negative solution that fits within the constraints of the system, the “+” solution might be discarded to avoid unphysical results. By simplifying the situation, *x* must be positive and less than both [D] and [P]. The term involving subtraction () typically results in the physically relevant concentration of the DNA–protein complex, *x*. Therefore, the bound fraction is then given by
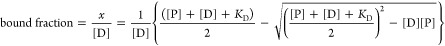
2

The third model incorporates cooperative binding properties, characterized by the Hill coefficient (*n*).
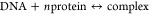


Given the dissociation equilibrium constant definition, *K*_D_ can be expressed as^22^
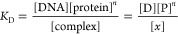


By substituting the definition of *K*_D_ into the bound fraction equation, it can be reformulated as
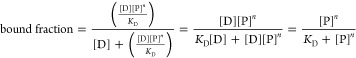
3

## Results and Discussion

### Rationale of Protein-Induced Fluorescence Change to Investigate Binding Affinity

In this study, a confocal microscopy-based approach for detecting protein-induced fluorescence change will be employed to determine the binding behaviors of HDGF, its mutants, and truncation domains. To verify the feasibility of using a confocal system to determine protein–nucleic acid-binding events based on protein-induced fluorescence change, a control experiment was conducted. It is known that *E. coli* RecA can bind to ssDNA to form a RecA nucleoprotein filament under physiological conditions. Moreover, a significant fluorescence increase can be observed when RecA proteins bind to Cy3-labeled ssDNA.^19,23^ A control experiment involving the RecA binding process was performed and recorded (Figure S2A). A significant fluorescence enhancement was observed, approaching a stable plateau at an *E. coli* RecA concentration of 100 nM [Figure S2B(i)]. The bound fractions were plotted against the concentration of *E. coli* RecA and fitted with an approximation single-site binding model. An apparent binding equilibrium constant, *K*_D_, of 34.5 ± 6.8 nM was obtained (Table S1), consistent with previously reported values^24^ (detailed discussion in Supporting Information), indicating that the confocal system-based PIFE is sensitive enough to determine protein binding properties. Therefore, 1 nM Cy3-labeled 15 bp *SMYD1* dsDNA (TTCAAGACCAGCCTG) was preincubated with HDGF at concentrations ranging from 10 pM to 100 nM on ice for 1 h before acquiring fluorescence signals (Figure 1A). Contrary to an expected increase in fluorescence intensity, a decrease in Cy3 fluorescence was observed after HDGF binding to 15 bp *SMYD1* dsDNA [Figure 1B(i), solid square]. Moreover, this decrease in fluorescence is proportional to the concentration of HDGF and reaches a plateau at low fluorescence intensities beyond a concentration of 10 nM. The binding fraction, normalized against the fluorescence decrease at 100 nM HDGF, was plotted against the HDGF concentration [Figure 1B(i), empty square]. Conversely, when assessing the PWWP domain’s binding to 15 bp *SMYD1* dsDNA, a noticeable, albeit slower, reduction in Cy3 fluorescence was recorded [Figure 1C(i), solid square]. In a recent study, Jarmoskaite et al. reported a guideline to correctly determine binding affinity of nucleic acid binding proteins.^25^ The single-site binding model approximation is valid when the protein is in substantial excess over the DNA in the experiment, implying that only a small fraction of the total protein added is bound to the DNA. However, when the contribution of the bound fraction *x* is significant, a more intricate quadratic binding equation form, or a nonapproximation single-site binding model, is employed for more accurate description of the binding behavior. It remains uncertain whether the interaction between HDGF and 15 bp *SMYD1* dsDNA is characterized by single- or multiple-site binding modes. To address these uncertainties, we employed three distinct models: the approximation model, the nonapproximation single-site binding model, and a model incorporating cooperative binding properties, quantified using the Hill coefficient (*n*). These models were used to analyze binding profiles generated using three different concentrations of 15 bp *SMYD1* dsDNA, under varying concentrations of HDGF and the PWWP domain of HDGF.

Experiments were systematically conducted by varying the labeled 15 bp *SMYD1* dsDNA concentration in HDGF/PWWP binding assays. It was noteworthy that the binding curve for HDGF could only be fitted to the approximation model, which had an adjusted *R*-square value of approximately 0.8. It could not be fitted to the nonapproximation model, which had an adjusted *R*-square value of less than −0.8, as shown in Figure 1B(ii) and Table 1. Varying labeled 15 bp *SMYD1* dsDNA concentrations in HDGF binding experiments revealed different binding profiles, indicating a dependence on apparent affinity with determined *K*_D_ values of 0.017, 0.019, and 0.031 nM by fitting to the approximation model for HDGF at 1, 5, and 10 nM 15 bp *SMYD1* dsDNA concentrations, respectively (Table 1). These results indicate that the approximation model is not suitable to interpret the binding behaviors of HDGF to 15 bp *SMYD1* dsDNA according to the guidelines reported by Jarmoskaite et al.^25^ Moreover, the determined *K*_D_ was significantly lower than the concentration of 15 bp *SMYD1* dsDNA, warranting further investigation into potential cooperativity between HDGF and 15 bp *SMYD1* dsDNA. Nonlinear least-squares fitting of the binding data to a cooperative binding model (Hill equation) resulted in apparent dissociation equilibrium constants (*K*_D_) of 0.095 ± 0.016 nM, 0.085 ± 0.024 nM, and 0.076 ± 0.020 nM for HDGF in the presence of 1, 5, and 10 nM 15 bp *SMYD1* dsDNA, respectively [Figure 1B(iii) and Table 1, with adjusted *R*-square values >0.8]. These *K*_D_ values were consistent across the tested 15 bp *SMYD1* dsDNA concentration range and were well-described by the cooperative binding model. The determined Hill coefficients were less than 1, implying that one HDGF might bind to more than one *SMYD1* DNA molecule, a 15 bp dsDNA with the sequence 5′-TTCAAGACCAGCCTG used here. Thus, the *K*_D_ value of approximately 0.1 nM, derived from the cooperative binding model, represents an estimated dissociation equilibrium constant for HDGF’s interaction with 15 bp *SMYD1* dsDNA under our experimental conditions.

In contrast, PWWP binding curves for 1 nM and 5 nM 15 bp *SMYD1* dsDNA concentrations were consistent, and the data were well-described by the approximation model, yielding nearly identical *K*_D_ values of approximately 1 nM [Figure 1C(ii,iii) and Table 1]. These findings suggest that the approximation model accurately describes the binding behavior of the PWWP domain to 15 bp *SMYD1* dsDNA. However, a slightly higher *K*_D_ value of 1.6 ± 0.2 nM was observed for the PWWP domain in the presence of 10 nM 15 bp *SMYD1* dsDNA by the approximation model. At 10 nM 15 bp *SMYD1* dsDNA, the data fits to all three models were less accurate, indicating a depletion of the PWWP domain due to its binding to labeled 15 bp *SMYD1* dsDNA. This suggests that the contribution of the bound fraction *x* cannot be ignored, and a reliable equilibrium constant can only be determined at a concentration of 1 nM 15 bp *SMYD1* dsDNA for the PWWP domain. For the PWWP domain binding to 15 bp *SMYD1* dsDNA, Hill coefficients of 1.00 were consistently obtained across all tested conditions, indicating noncooperative binding behavior. Consequently, the *K*_D_ values determined for the PWWP domain by fitting data to the approximation, nonapproximation, and cooperative binding models were insignificantly different, affirming them as reliable equilibrium constants (Table 1, with adjusted *R*-square values of >0.96). The *K*_D_ values displayed in Table 1 were obtained by using three different models, each with an adjusted *R*-square value greater than 0.8. Fitting results that produced adjusted *R*-square values less than 0 are marked with “N.F.”, signifying that the data could not be fitted. Cells containing a diagonal line denote a lack of detectable signals, indicated by “N.D.”, which stands for not detectable. For clarity in comparative discussions, subsequent discussions will focus on *K*_D_ values determined from the cooperative binding model (presented in bold in Table 1).

**Figure 2:**
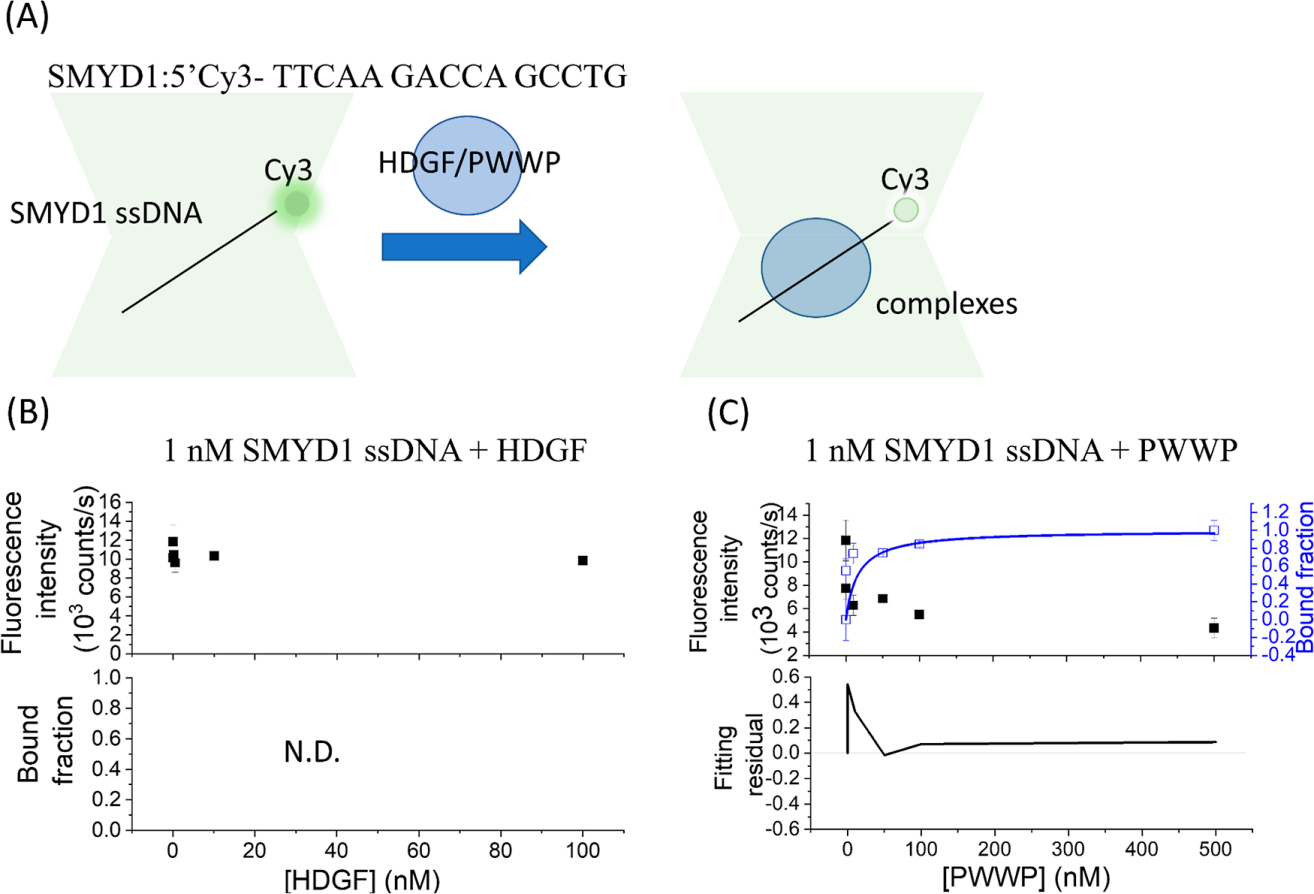
(A) Schematic of HDGF binding to a Cy3-labeled ssDNA signaled by PIFQ. (B) PIFQ-based measurement of binding affinity of HDGF to 1 nM *SMYD1* ssDNA (*K*_D_ = N.D.). (C) PIFQ-based measurement of binding affinity of the PWWP domain to 1 nM *SMYD1* ssDNA (*K*_D_ = 16 ± 3 nM). The solid circles indicate the fluorescence intensity. The open squares indicate the normalized bound fraction. The solid lines represent the fitting curves with the approximation model to obtain corresponding dissociation equilibrium constants, *K*_D_, listed in Table 1.

A similar experimental approach with ssDNA revealed no significant change in fluorescence, indicating HDGF’s preference for dsDNA (Figure 2B), which is consistent with previous reports.^13^ Interestingly, a significant decrease in fluorescence intensity was observed for 1 nM Cy3-labeled 15 nt *SMYD1* ssDNA preincubated with the PWWP domain, resulting in an apparent *K*_D_ of 16 ± 3 nM (Figure 2C and Table 1). This finding implies that the PWWP domain of HDGF can also bind to ssDNA, consistent with previous reports.^13^

**Figure 3:**
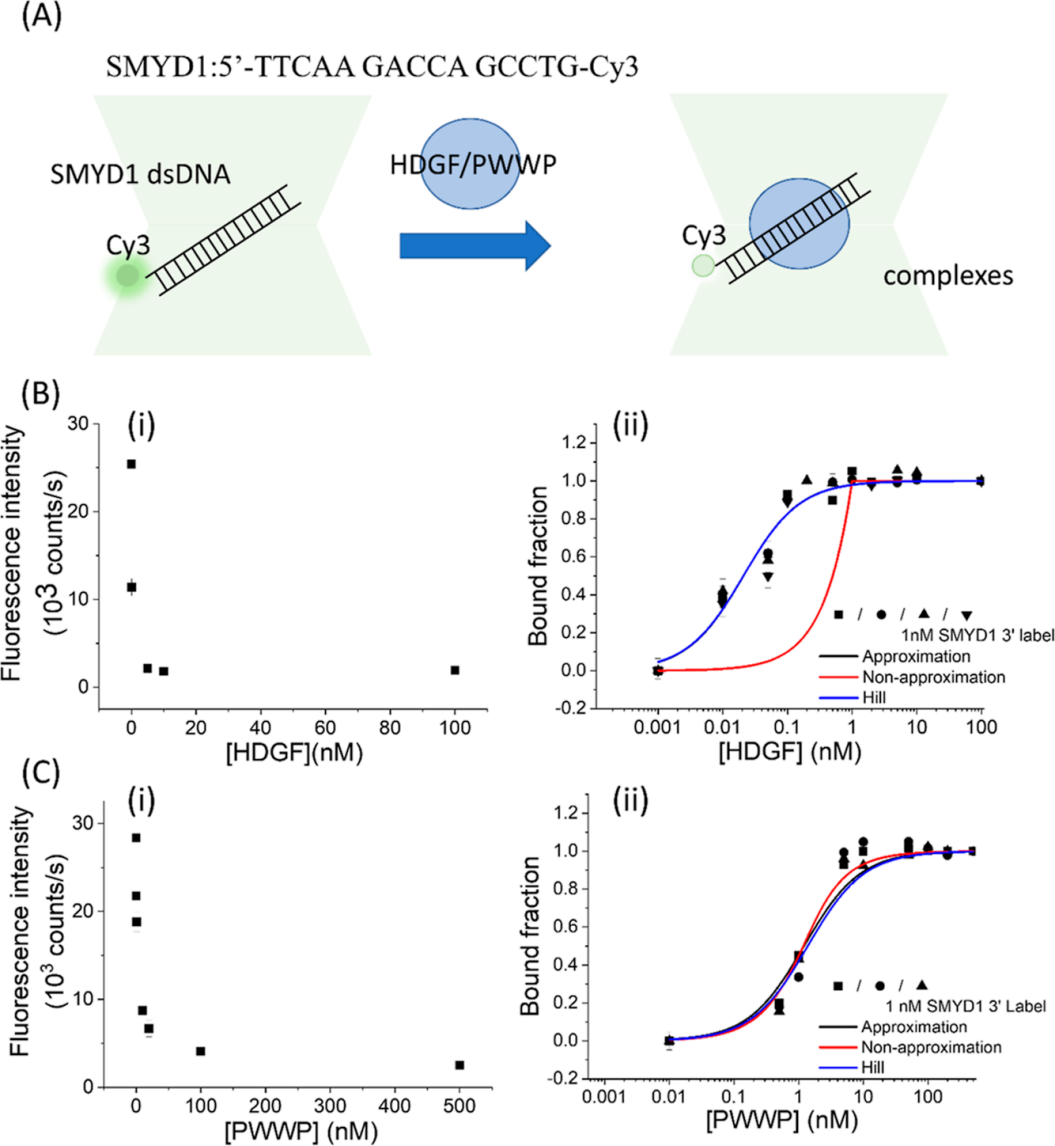
Protein-induced fluorescence change to investigate binding affinity of HDGF and the PWWP domain to SMYD1 dsDNA with Cy3 labeled in the 3′ end. (B) PIFQ-based measurement of binding affinity of HDGF to 1 nM *SMYD1*. (i) Decrease in Cy3 fluorescence intensity after adding HDGF. (ii) The bound fraction is fitted to the Hill equation to obtain an apparent *K*_D_ of 0.080 ± 0.004 (*n* = 0.60). (C) PIFQ-based measurement of binding affinity of the PWWP domain to 1 nM *SMYD1*. (i) Decrease in Cy3 fluorescence intensity after adding PWWP. (ii) The bound fraction is fitted to the Hill equation to obtain an apparent *K*_D_ of 1.4 ± 0.2 (*n* = 1.0). The lines represent the fitting curves with the Hill equation fitting model (blue: —), approximation fitting model (black: —), and nonapproximation model (red: —) to obtain corresponding dissociation equilibrium constants, *K*_D_, listed in Table 1. (-■-, -●-, -▲-, and -▼- indicate data from different repeated experiments.)

Regarding hRPA’s interaction with Cy3-labeled ssDNA, significant fluorescence change was observed, which varied depending on the fluorophore’s labeling position.^26^ Moreover, Rashid et al. have reported that the initial fluorescence state of the labeled mediator (DNA) determines whether the mediator-conjugated dye undergoes PIFE or PIFQ.^20^ Our study further compares Cy3 fluorescence changes when HDGF/PWWP binds to 15 bp *SMYD1* dsDNA with Cy3 labeled at the 3′ end (Figure 3). We observed a notable decrease in fluorescence intensity upon protein binding, with similar apparent *K*_D_ values, indicating that the labeling position does not significantly influence HDGF's/PWWP’s binding behavior to 15 bp *SMYD1* dsDNA (Table 1).

### PWWP Domain Is Required for DNA Binding and the C140 Domain Can Enhance the DNA Binding Affinity

**Figure 4:**
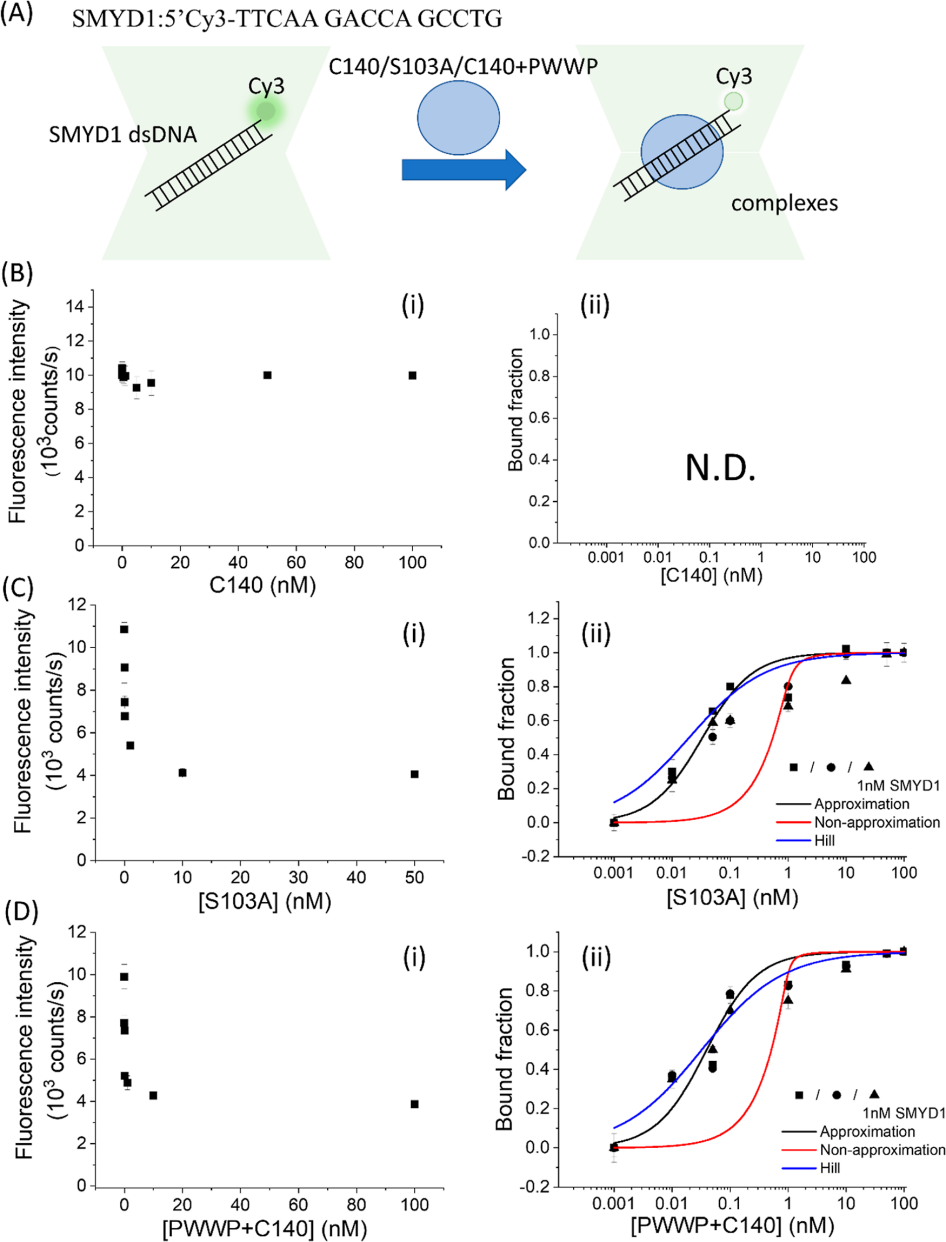
PIFQ-based measurement of binding affinity of HDGF mutants to 1 nM 15 bp SMYD1 dsDNA. (B) Binding behavior of HDGF truncated mutant, C140 domain. (C) Binding behavior of HDGF point mutant, S103A. (D) Binding behavior of premixed HDGF truncated mutants, PWWP + C140. The lines represent the fitting curves with the Hill equation fitting model (blue: —), approximation fitting model (black: —), and nonapproximation model (red: —) to obtain corresponding dissociation equilibrium constants, *K*_D_, listed in Table 1.

HDGF comprises PWWP and C140 domains, with the PWWP domain being essential for DNA binding.^10^ The N-terminal domain of HDGF is highly conserved, but the C-terminal 140 residue (C140) domain is variable. It is interesting to investigate the function of the C140 domain, and determine whether it influences the DNA-binding behavior of HDGF. Moreover, the S103A mutant of HDGF has been reported to lose its ability to mediate cell invasion and proliferation.^12^ It would be valuable to explore whether the dysfunction induced by the S103A mutation is due to altered DNA binding of HDGF to *SMYD1* dsDNA. Consequently, we are employing the PIFQ technique to assess the binding behavior of the C140 domain and S103A mutants in comparison to the HDGF and PWWP domain. In this experiment, the Cy3 fluorophore was labeled at the 5′ end of 15 bp *SMYD1* dsDNA and titrated with HDGF mutants to observe changes in fluorescence intensity (Figure 4). Initially, the C140 domain, at concentrations ranging from 0.001 to 100 nM, was preincubated with 1 nM Cy3-labeled 15 bp *SMYD1* dsDNA. No significant decrease in the fluorescence intensity of the Cy3 fluorophore was observed following the addition of the C140 domain. This indicates the absence of detectable binding between the C140 domain and 15 bp *SMYD1* dsDNA, reinforcing the assertion that the PWWP domain is necessary for DNA binding (Figure 4A). However, upon the addition of the HDGF mutant S103A, a significant decrease in the fluorescence intensity of the Cy3 fluorophore was observed. The plateau value observed for DNA in response to S103A is slightly smaller than that observed with HDGF and the PWWP domain. This difference might result from the different binding modes among these proteins, which cause slight variations in the quantum yield of Cy3. The binding data were fitted to the Hill equation (Figure 4B), yielding an apparent *K*_D_ of 0.072 ± 0.003 nM with a Hill coefficient of 0.67. This *K*_D_ value is similar to that of HDGF, suggesting that the serine-to-alanine mutation does not significantly affect binding affinity to 15 bp *SMYD1* dsDNA. It is intriguing to explore whether adding the C140 domain to the PWWP domain will enhance the binding behavior, making it more similar to that of full-length HDGF. When equal amounts of the C140 domain and PWWP domain were preincubated for half an hour before being added to 1 nM Cy3-labeled 15 bp *SMYD1* dsDNA, a significant decrease in Cy3 fluorescence intensity was observed (Figure 4C). A smaller plateau value was also observed for DNA in response to the premixed C140 domain and PWWP domain suggesting the presence of different binding modes causing slight variations in the quantum yield of Cy3. Fitting the bound fraction to the Hill equation resulted in an apparent *K*_D_ of 0.12 ± 0.04 nM with a Hill coefficient of 0.63 (Figure 4C and Table 1). The apparent *K*_D_ for the mixture of the C140 domain and PWWP domain is lower than that for the PWWP domain alone and is comparable to that of full-length HDGF. This observation suggests that the variable C-terminal domain in HDGF may enhance DNA-binding capability.

### C140 Domain Is Crucial for Specific Sequence DNA Binding

**Figure 5:**
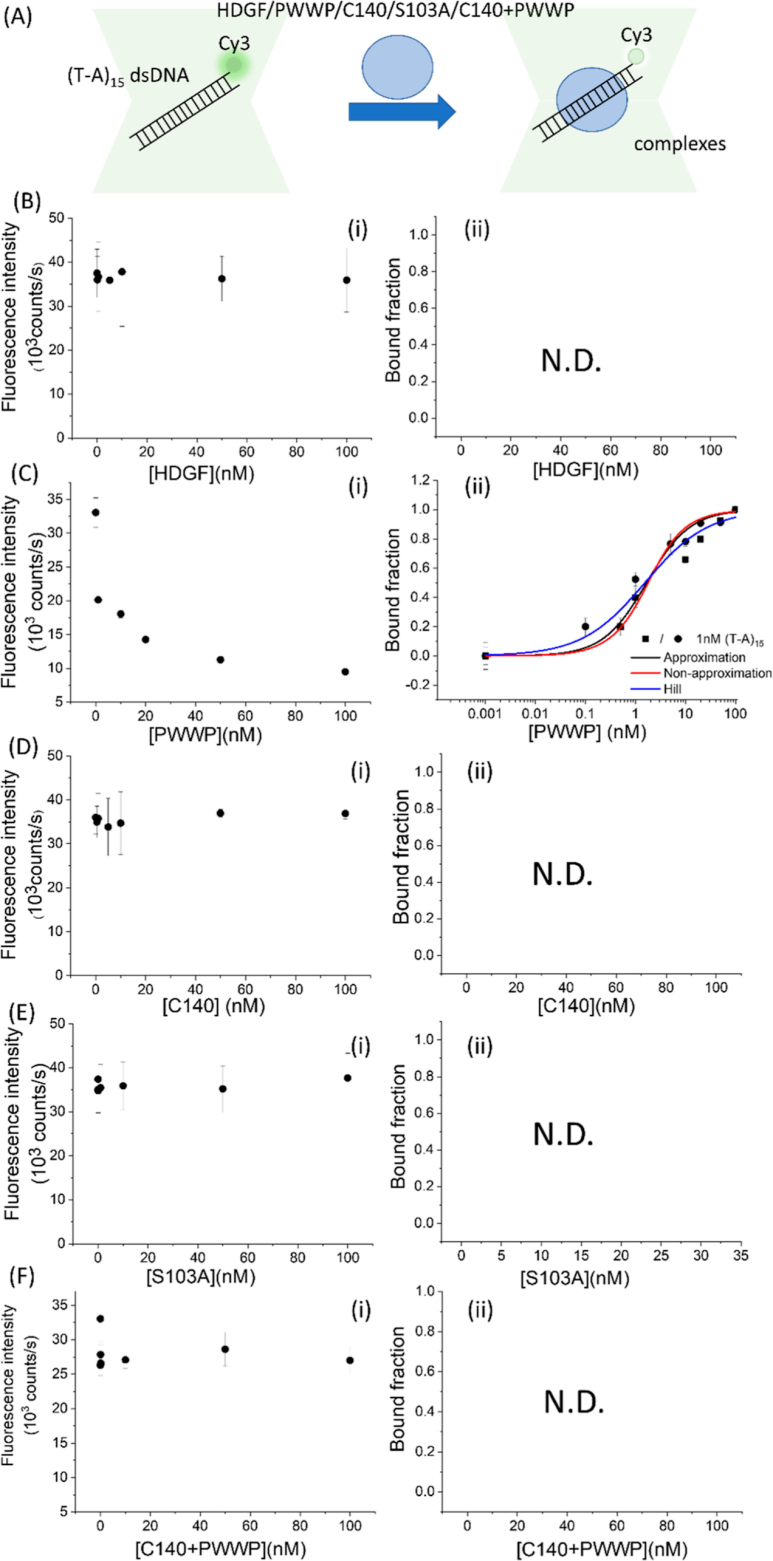
PIFQ-based measurement of binding affinity of HDGF and mutants to 1 nM (T-A)_15_ dsDNA. (B) Binding behavior of HDGF. (C) Binding behavior of the PWWP domain. (D) Binding behavior of HDGF truncated mutant, C140 domain. (E) Binding behavior of HDGF point mutant, S103A. (F) Binding behavior of premixed HDGF truncated mutants, PWWP + C140. The lines represent the fitting curves with the Hill equation fitting model (blue: —), approximation fitting model (black: —), and nonapproximation model (red: —) to obtain corresponding dissociation equilibrium constants, *K*_D_, listed in Table 1.

DNA-binding assays have shown that the PWWP domain of HDGF does not exhibit a sequence specificity preference for dsDNA.^13^ However, HDGF has been demonstrated to specifically bind to the *SMYD1* promoter, functioning as a transcriptional repressor.^10,27^ While HDGF interacts with the *SMYD1* promoter through its N-terminal PWWP domain, the detailed mechanisms regulating its sequence specificity in binding to *SMYD1* remain unclear.^10^ In our PIFQ experiment, we found that the PWWP domain is essential for DNA binding, while the C140 domain significantly enhances this binding affinity (Figure 4 and Table 1). For this study, we labeled the Cy3 fluorophore at the 5′ end of poly(T-A)_15_ dsDNA and titrated it with HDGF and related mutants to investigate DNA sequence specificity (Figure 5). A noticeable decrease in Cy3 fluorescence intensity was observed solely in interactions between Cy3-labeled poly(T-A)_15_ dsDNA and the PWWP domain (Figure 5C). The binding data for the PWWP domain with poly(T-A)_15_ dsDNA fit a cooperative binding model (Table 1), revealing a 1.7-fold lower affinity for PWWP binding to poly(T-A)_15_ dsDNA, with an apparent *K*_D_ of 1.6 ± 0.2 nM (Figure 5C). This suggests a slightly weaker binding affinity to poly(T-A)_15_ dsDNA compared to 15 bp *SMYD1* dsDNA for the PWWP domain (Figures 1C and 5C and Table 1). Moreover, no significant decrease in Cy3 fluorescence intensity was observed when poly(T-A)_15_ dsDNA was preincubated with HDGF or its mutants, the C140 domain, and S103A, indicating that they do not bind to poly(T-A)_15_ dsDNA (Figure 5E). Subsequent PIFQ experiments with Cy3-labeled poly(T-A)_15_ dsDNA titrated with a premixed solution of the C140 domain and the PWWP domain showed a slight decrease in fluorescence intensity upon the addition of the premixed proteins (Figure 5F). However, this decrease was minimal and remained consistent, even after the addition of higher concentrations of the premixed protein. This observation suggests that the modest decrease in fluorescence, indicating weak DNA binding, is likely due to the nonspecific DNA-binding behavior of the PWWP domain, which does not interact significantly with the C140 domain. These experimental results imply that while the C140 domain does not directly bind to DNA, it can enhance the DNA sequence specificity of the PWWP domain of HDGF through protein–protein interactions (Table 1).

**Figure 6:**
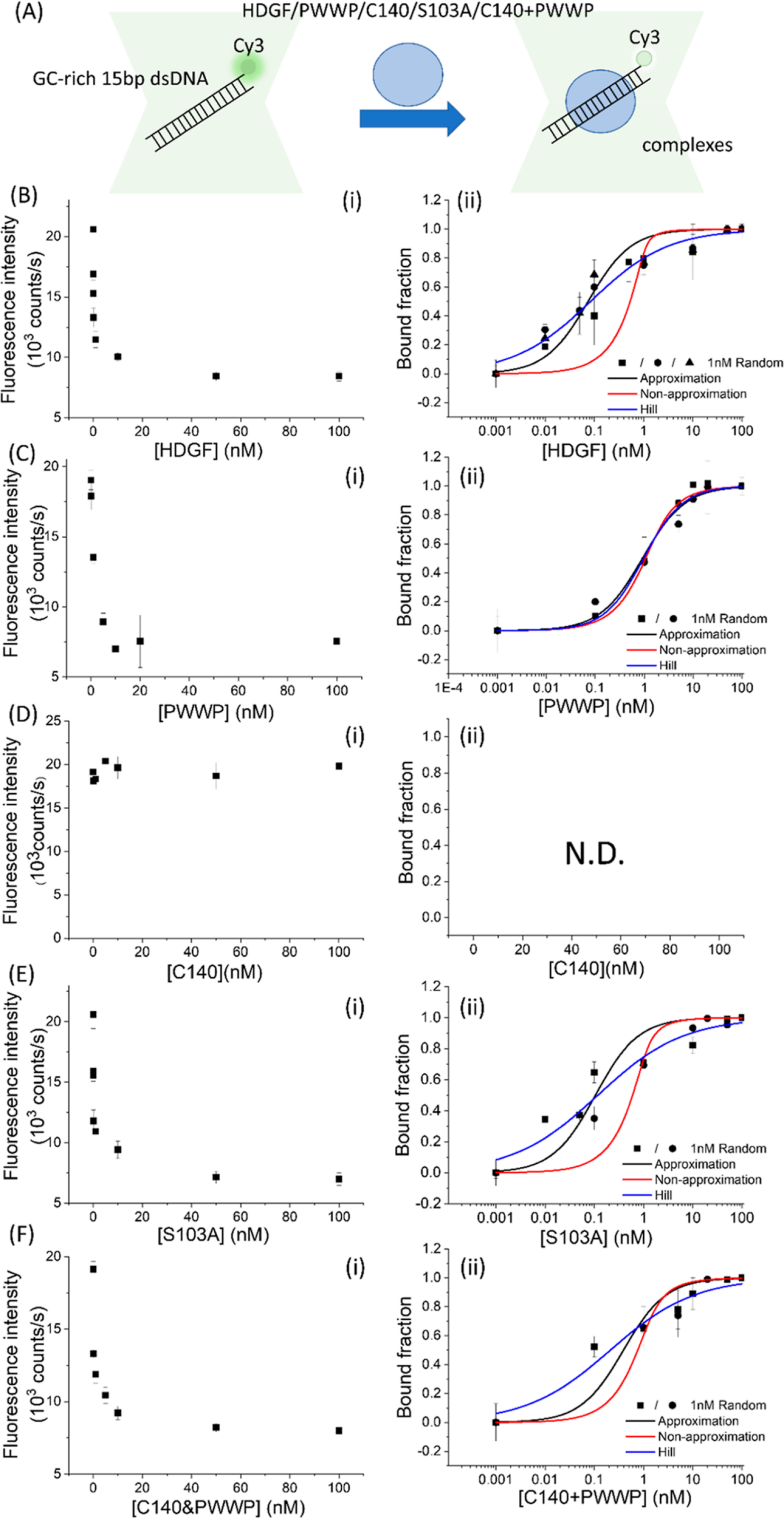
PIFQ-based measurement of binding affinity of HDGF and mutants to 1 nM 15 bp GC-rich dsDNA. (B) Binding behavior of HDGF. (C) Binding behavior of the PWWP domain. (D) Binding behavior of HDGF truncated mutant, C140 domain. (E) Binding behavior of HDGF point mutant, S103A. (F) Binding behavior of premixed HDGF truncated mutants, PWWP + C140. The lines represent the fitting curves with the Hill equation fitting model (blue: —), approximation fitting model (black: —), and nonapproximation model (red: —) to obtain corresponding dissociation equilibrium constants, *K*_D_, listed in Table 1.

A previous study reported that the PWWP domain of HRP3 shows a stronger binding preference for TA-rich DNA molecules over GC-rich ones.^15^ TA-rich DNA is more suitable for the PWWP domain of HRP3 binding, while GC-rich DNA, characterized by a wider minor groove, requires narrowing for efficient interaction with this domain.^15^ Thus, we selected Cy3-labeled GC-rich dsDNA molecules with the sequence (5′-TCCTCGCTGCCGTCGGCCA-3′, GC % = 78%) to investigate the sequence preference of HDGF and its mutants (Figure 6A). In contrast to TA-rich DNA molecules [poly(T-A)_15_ dsDNA, Figure 5], a detectable decrease in Cy3 fluorescence intensity was observed for all investigated proteins except the C140 domain on GC-rich dsDNA molecules (Figure 6). This finding reinforces the essential role of the PWWP domain in DNA binding.^10^ All binding data were fitted with the Hill equation, yielding apparent *K*_D_ values of 0.25 ± 0.03 nM (*n* = 0.55), 0.97 ± 0.19 nM (*n* = 1.00), 0.34 ± 0.06 nM (*n* = 0.50), and 0.45 ± 0.06 nM (*n* = 0.50) for HDGF, the PWWP domain, S103A, and a premixed equal amount of the PWWP domain and C140 domain, respectively (Figure 6 and Table 1). Compared to its binding behavior with 15 bp *SMYD1* dsDNA (Figure 1B and Table 1), HDGF binds to 15 bp GC-rich dsDNA with approximately 3-fold weaker affinity. This contrasts with the lack of binding to TA-rich dsDNA (Figure 5B), suggesting that the high GC content in the 15 bp *SMYD1* dsDNA may be one of the factors contributing to HDGF’s higher affinity. Similarly, the decreased dsDNA binding affinity of S103A on GC-rich dsDNA, compared to that of 15 bp *SMYD1* dsDNA, indicates that the sequence specificity is not affected by the mutation at residue 103. However, PWWP binds to 15 bp GC-rich dsDNA and 15 bp *SMYD1* dsDNA with comparable affinity (*K*_D,GC-rich_ = 0.97 ± 0.19 nM vs *K*_D,*SMYD1*_ = 0.94 ± 0.10 nM, Table 1) but binds to 15 bp TA-rich dsDNA with approximately twofold weaker affinity (*K*_D,TA-rich_ = 1.6 ± 0.2 nM, Table 1). This suggests that although the PWWP domain binds DNA nonspecifically,^7,13^ it still exhibits a slightly stronger binding preference for GC-rich dsDNA molecules over TA-rich ones. Interestingly, premixing equal amounts of the C140 domain with the PWWP domain enhances the binding affinity of the PWWP domain to 15 bp GC-rich dsDNA approximately twofold (*K*_D,GC-rich,PWWP_ = 0.97 ± 0.19 nM vs *K*_D,GC-rich,PWWP+C140_ = 0.45 ± 0.06 nM, Table 1). This highlights the regulatory role of the C140 domain in the DNA binding affinity of the PWWP domain. Based on the results from EMSAs, it has been confirmed that HDGF binds to an 80 bp conserved sequence located in the SMYD1 promoter.^10^ This 80 bp dsDNA is situated at positions −688 to −609 of the SMYD1 promoter (+1 being the start codon), with the sequence 5′-CAGGCTGGTCTTGAACTCCTGACCTCAGATGATCCATGTGCCTCGGCCTCCCAAGGTGGGGATTACAGGCGTGAGCCACC-3′. The GC content in this region is 60%, implying a preferential binding of HDGF to regions with high GC content, which corroborates our findings from PIFQ analysis. These observations support the hypothesis that HDGF preferentially binds to GC-rich dsDNA over TA-rich dsDNA, a process influenced by the C140 domain. However, further experiments should investigate variations within the SMYD promoter to validate this hypothesis.

## Conclusions

Our study utilizing PIFQ has provided significant insights into the DNA-binding characteristics of HDGF and its domains, shedding light on their roles in cellular functions. This study particularly highlights the binding preferences and mechanisms of the PWWP domain in HDGF, as well as the influential role of the C140 domain in modulating DNA binding affinity and sequence specificity. The nonspecific DNA binding affinity of the PWWP domain in HDGF, as established through NMR titration^13^ and PIFQ experiments, contrasts with HDGF’s specific binding to the *SMYD1* promoter.^10^ This specificity is crucial for HDGF’s function as a transcriptional repressor,^27^ yet the detailed mechanisms underlying this specificity remain elusive. Our findings demonstrate that while the PWWP domain is essential for DNA binding, the C140 domain significantly enhances this binding affinity. Moreover, when investigating sequence preferences, our study revealed that HDGF and its mutants exhibit a stronger binding affinity for GC-rich dsDNA over TA-rich dsDNA. This preference, also regulated by the C140 domain, suggests a regulation mechanism in which the C140 domain does not directly bind to DNA but enhances the sequence specificity of the PWWP domain through protein–protein interactions. These findings are in line with previous studies indicating the nonspecific binding nature of the PWWP domain in various proteins^14,15,28^ but full length HDGF protein with the PWWP domain and C140 domain exhibits sequence-specific DNA-binding behavior.^10^

Our study employed three different models to analyze binding profiles, including the approximation model, the nonapproximation single-site binding model, and a model incorporating cooperative binding properties. The consistency of *K*_D_ values obtained from the cooperative binding model across different 15 bp *SMYD1* dsDNA concentrations confirmed it as a more reliable description of HDGF’s binding behavior, suggesting the potential cooperativity between HDGF and *SMYD1*. Moreover, the study’s findings on the PWWP domain’s binding behaviors offer a comprehensive view of its interaction with dsDNA and ssDNA. The domain’s comparable affinity for both GC-rich and *SMYD1* dsDNA, but weaker affinity for TA-rich dsDNA, highlights its generally nonspecific binding nature, which is subtly influenced by sequence context.

HDGF’s function as a transcriptional repressor is primarily mediated through its binding to specific DNA sequences, such as the *SMYD1* promoter.^10^ The PWWP domain of HDGF, essential for DNA binding, exhibits nonspecific interactions with DNA, as established through NMR titration.^13^ However, our PIFQ experiments reveal a nuanced behavior where the S103A mutation does not significantly affect HDGF’s binding affinity to 15 bp *SMYD1* dsDNA, as evidenced by the similar *K*_D_ values obtained for both wild-type HDGF and the S103A mutant. The S103A mutation has been previously reported to impair HDGF-mediated cell invasion and proliferation.^12^ Our findings suggest that this impairment is not due to a loss of DNA binding affinity per se but potentially results from altered interactions with other molecular components involved in the gene regulation pathways. This observation is critical in understanding the mechanistic aspects of HDGF’s role in cancer progression.

A previous study demonstrated that PWWP-10 bp *SMYD1* dsDNA complexes exist as dimers, as confirmed by size calibration chromatography.^4^ It has also been reported that the apo form of the PWWP domain predominantly exists as a monomer at initial concentrations below ∼1.5 mg/mL (∼0.15 mM), with the proportion of dimers increasing at higher concentrations. Moreover, it was noted that the dimeric PWWP domain transitions into monomers within 3 days in buffer solutions containing ionic-strength salts such as NaCl (150 mM).^4^ Under our experimental conditions (<0.5 μM of PWWP), the presence of the dimeric PWWP domain is considered negligible. Consequently, the binding behaviors described in our study are focused solely on the interaction between the monomeric PWWP domain and the DNA substrate.

Regarding hHDGF, the binding behaviors have been discussed in the following two papers. The first study by Lukasik et al. suggests that the PWWP domain of HDGF may function as a nonspecific DNA-binding domain. This was determined using NMR titrations and a combination of NOEs, J couplings, and dipolar couplings to ascertain the NMR structure of the HDGF PWWP domain.^13^ The second study by Yang and Everett identifies the N-terminal PWWP domain of HDGF as essential for DNA binding, mapping the functional DNA-binding domain and element using ChIP and the EMSA.^10^ Neither of the studies reported the dissociation constant (*K*_D_) for either hHDGF or its PWWP domain. So far, the *K*_D_ of the PWWP domain of the human mismatch repair protein MSH6 has been investigated by the EMSA to examine binding behaviors and to determine a dissociation constant (*K*_D_) of 5.64 nM, similar to PWWP’ *K*_D_ obtained from the PIFQ system in our laboratory.^14^

In conclusion, our study not only elucidates the complex interaction behaviors of HDGF and its domains with DNA but also provides a foundation for future research into molecular mechanisms underlying HDGF’s regulation of cellular functions. The interplay between the PWWP domain and C140 domains in determining DNA-binding specificity could pave the way for new therapeutic strategies targeting HDGF’s role in tumor growth and metastasis.

## Supporting Information Available

Depiction and SDS-PAGE analysis of HDGF and its related domains, PIFE data of interactions between DNA and *E. coli**RecA* and *Deinococcus ficus**RecA*, and dissociation equilibrium constants of *E. coli* RecA and *D. ficus* RecA to ssDNA and dsDNA molecules (PDF)
