# Supplementary material for: Binding Behavior of Human Hepatoma-Derived Growth Factor on SMYD1
Source: J Phys Chem B. 2024 Aug 2;128(32):7722–35. doi: 10.1021/acs.jpcb.4c01854 (PMC11331505; doi:10.1021/acs.jpcb.4c01854)
Supplement: Supplementary file 1 — jp4c01854_si_001.pdf [file jp4c01854_si_001.pdf]

Supporting Information

Binding Behavior of Human Hepatoma-Derived Growth Factor on *SMYD1*

Jan-Kai Wu[1 2.3], Ying-ying Lee [1 2.3], Hsin Hung [1 2.3], Yuan-Ping Chang[1,3], Ming-Hong Tai[4], Hsiu-Fang Fan[1 2.3*]

[1] Institute of Medical Science and Technology, National Sun Yat-sen University, 80424, Taiwan

[2] Department of Chemistry, National Sun Yat-sen University, 80424, Taiwan

[3] Aerosol Science Research Center, National Sun Yat-sen University, 80424, Taiwan

[4] Institute of Biomedical Science, National Sun Yat-sen University, 80424, Taiwan

Supplementary information:

Here, 1 nM Cy3-labeled poly $(dT)_{40}$ ssDNA was preincubated with *E. coli* RecA at concentrations ranging from 1 nM to 500 nM on ice for 1 hour before fluorescence acquisition. A significant fluorescence enhancement was observed, approaching a stable plateau at *E. coli* RecA concentration of 100 nM (Figure S2B (i)). Normalized to the fluorescence increase in the presence of 500 nM *E. coli* RecA, the bound fractions were plotted against the concentration of *E. coli* RecA and fitted with an approximation single-site binding model. An apparent binding equilibrium constant, K_D, of 34.5 ± 6.8 nM was obtained, consistent with previously reported values [1]. Furthermore, the confocal system-based protein-induced fluorescence enhancement (PIFE) was used to investigate the DNA binding properties of *D. ficus* RecA. A significant increase in fluorescence intensity was also observed for 1 nM Cy3-labeled poly $(dT)_{40}$ ssDNA preincubated with *D. ficus* RecA proteins (Figure S2B (ii)), and apparent binding equilibrium constants, K_D, of 13.6 ± 1.4 nM was obtained (Table S1). However, there was no detectable change in fluorescence for 1 nM Cy3-labeled poly $(T\text{-}A)_{40}$ dsDNA preincubated with *E. coli* RecA, indicating a weak binding affinity on dsDNA at pH 7.5 (Figure S2C and Figure S2D (i)), consistent with previous studies [2]. In contrast, a similar fluorescence increase trend was observed for 1 nM Cy3-labeled poly $(T\text{-}A)_{40}$ dsDNA preincubated with *D. ficus* RecA, and an apparent binding equilibrium constant, K_D, of 19.0 ± 7.0 nM was obtained, indicating a strong dsDNA binding affinity for *D. ficus* RecA compared to *E. coli* RecA (Figure S2D (ii)), consistent with previous studies[2].

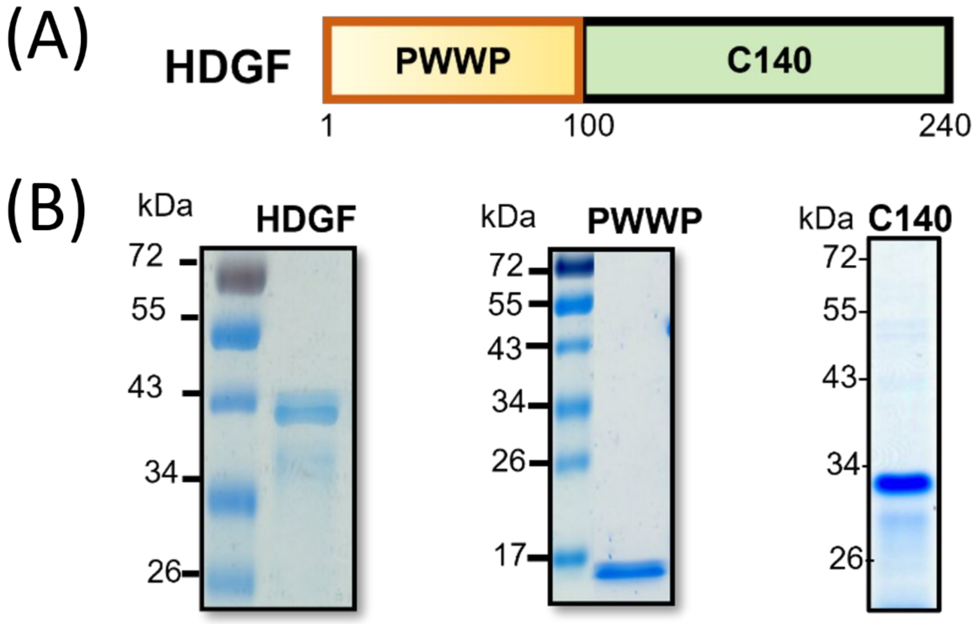

Figure S1 A. Schematic depiction of hepatoma-derived growth factor (HDGF) and its component PWWP domain (residues 1 to 100) and C140 domain (residues 101 to 240). B. SDS-PAGE analysis of recombinant HDGF, PWWP, and C140 domain expressed and purified from *E. coli*.

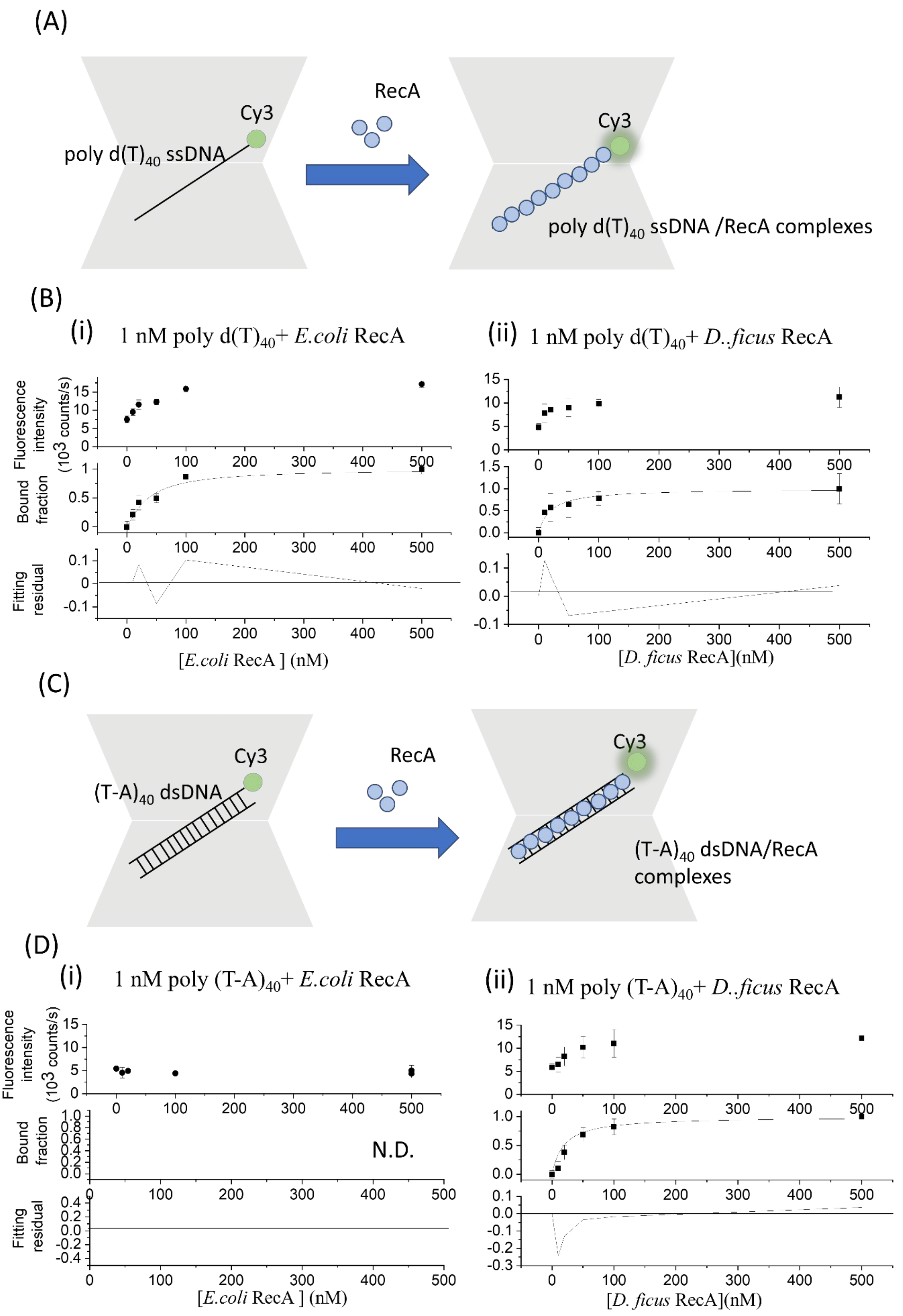
(A)
Cy3
poly d(T)40 ssDNA
RecA
Cy3
poly d(T)40 ssDNA /RecA complexes
(B)
(i) 1 nM poly d(T)40+ E.coli RecA
(ii) 1 nM poly d(T)40+ D..ficus RecA
Fluorescence intensity (10^3 counts/s)
Bound fraction
Fitting residual
[E.coli RecA] (nM)
[D. ficus RecA](nM)
(C)
Cy3
(T-A)40 dsDNA
RecA
Cy3
(T-A)40 dsDNA/RecA complexes
(D)
(i) 1 nM poly (T-A)40+ E.coli RecA
(ii) 1 nM poly (T-A)40+ D..ficus RecA
Fluorescence intensity (10^3 counts/s)
Bound fraction
N.D.
Fitting residual
[E.coli RecA] (nM)
[D. ficus RecA](nM)

Figure S2 *Protein-induced fluorescence changes to investigate DNA binding affinity of E. coli RecA and D, ficus RecA.* **A**. Schematic of RecA binding to a Cy3-labeled ssDNA signaled by significant fluorescence intensity enhancement (PIFE). **B.** (i) Fluorescence changes of 1nM Cy3-labeled poly $(dT)_{40}$ ssDNA in response to various concentration *E. coli* RecA, the corresponding bound fraction, and the fitting residues to an approximation of a single-site binding model. (ii) Fluorescence changes of 1nM Cy3-labeled poly $(dT)_{40}$ ssDNA in response to various concentration *D, ficus* RecA, the corresponding bound fraction, and the fitting residues to an approximation of a single-site binding model. **C.** Schematic of RecA binding to a Cy3-labeled dsDNA signaled by significant fluorescence intensity enhancement (PIFE). **D.** (i) Fluorescence changes of 1nM Cy3-labeled poly $(TA)_{40}$ dsDNA in response to various concentration *E. coli* RecA, the corresponding bound fraction, and the fitting residues to an approximation of a single-site binding model. (ii) Fluorescence changes of 1nM Cy3-labeled poly $(T\text{-}A)_{40}$ dsDNA in response to various concentration *D, ficus* RecA, the corresponding bound fraction, and the fitting residues to an approximation of a single-site binding model. Three repeated experiments were conducted to investigate the interaction between RecA and DNA fitted with three binding models. The obtained corresponding dissociation equilibrium constants, K_D, are listed in Table S1.

Supplementary Table 1. Dissociation equilibrium constants, K_D, of *E. coli* RecA and *D. ficus* RecA to ssDNA and dsDNA molecules. The rate constants were derived by fitting the data with an approximation-fitting model. N.D. means not detectable.

DNA Substrate		***Proteins***	***K_D Approximation*** (nM)
Name	***Concentration (nM)***		
$d(T)_{40}$ ssDNA	***1***	***E coli. RecA***	34.5 ± 6.8
	1	***D. Ficus RecA***	13.6 ± 1.4
$d(TA)_{40}$ dsDNA	***1***	***E coli. RecA***	***N.D.***
	1	***D. Ficus RecA***	19.0 ± 7.0

(1) Gataulin, D. V.; Carey, J. N.; Li, J.; Shah, P.; Grubb, J. T.; Bishop, D. K. The ATPase activity of E. coli RecA prevents accumulation of toxic complexes formed by erroneous binding to undamaged double stranded DNA. *Nucleic Acids Res* **2018**, *46* (18), 9510-9523. DOI: 10.1093/nar/gky748 From NLM Medline.
(2) Fan, H. F.; Su, S.; Kuo, Y. A.; Chen, C. J. Influence of the C-Terminal Tail of RecA Proteins from Alkaline pH-Resistant Bacterium Deinococcus Ficus. *ACS Omega* **2020**, *5* (31), 19868-19876. DOI: 10.1021/acsomega.0c02865. Fan, H. F.; Su, S. The regulation mechanism of the C-terminus of RecA

proteins during DNA strand-exchange process. *Biophys J* **2021**, *120* (15), 3166-3179. DOI: 10.1016/j.bpj.2021.06.004   From NLM Medline.
